# Measuring the effects of particle orientation to improve the efficiency of electron cryomicroscopy

**DOI:** 10.1038/s41467-017-00782-3

**Published:** 2017-09-20

**Authors:** Katerina Naydenova, Christopher J. Russo

**Affiliations:** 1 MRC Laboratory of Molecular Biology, Francis Crick Avenue, Cambridge, CB2 0QH UK; 2 Trinity College, University of Cambridge, Cambridge, CB2 1TQ UK

## Abstract

The orientation distribution of a single-particle electron cryomicroscopy specimen limits the resolution of the reconstructed density map. Here we define a statistical quantity, the efficiency, *E*_od_, which characterises the orientation distribution via its corresponding point spread function. The efficiency measures the ability of the distribution to provide uniform information and resolution in all directions of the reconstruction, independent of other factors. This metric allows rapid and rigorous evaluation of specimen preparation methods, assisting structure determination to high resolution with minimal data.

## Abstract (web summary)

A number of parameters influence the resolution of a cryo-EM structure. Here the authors investigate the effects of specimen orientation in single particle cryo-EM and present open-source software for rapidly assessing orientation distributions to improve data collection.

## Introduction

Recent technological advances have ushered in a new era of structure determination by single-particle electron cryomicroscopy (cryo-EM). Better electron detectors, new reconstruction algorithms and reduced movement of specimens during imaging, either through the use of software tracking algorithms or specimen supports, have all contributed to the rapid advance of the field^1–3^. Still, the specimen and how it interacts with the surfaces of the support can limit and even prevent structure determination.

Cryo-EM requires a thin specimen that is stable in the vacuum of the electron microscope. Specimen supports are designed to create and preserve thin films of water, which contain the purified biological sample^4^. During the cryo-plunging process, the purified particles can diffuse into contact with the surface of the thin film of water anywhere from 10 to more than 1000 times before freezing using current techniques (Supplementary Note 1)^5^. This gives ample opportunity for a particular region of the particle surface to adhere to the water surface, and thus induce a preferential orientation of the particle on the support. When imaged, micrographs are taken perpendicular to the surface of the thin film to provide highest contrast. So if the particles are not randomly oriented relative to the surface, this limits the available information and can prevent high-resolution three-dimensional (3D) structure determination.

Previous works addressing directional resolution in single-particle cryo-EM^6, 7^, and electron cryotomography^8^ have characterised the final reconstructed density maps in terms of missing regions of Fourier space. Here, we define a statistical quantity, the “efficiency” (*E*_od_), which characterises the specimen orientation distribution by its ability to provide uniform resolution in all directions.

## Results

### Analytical derivation

**Fig. 1:**
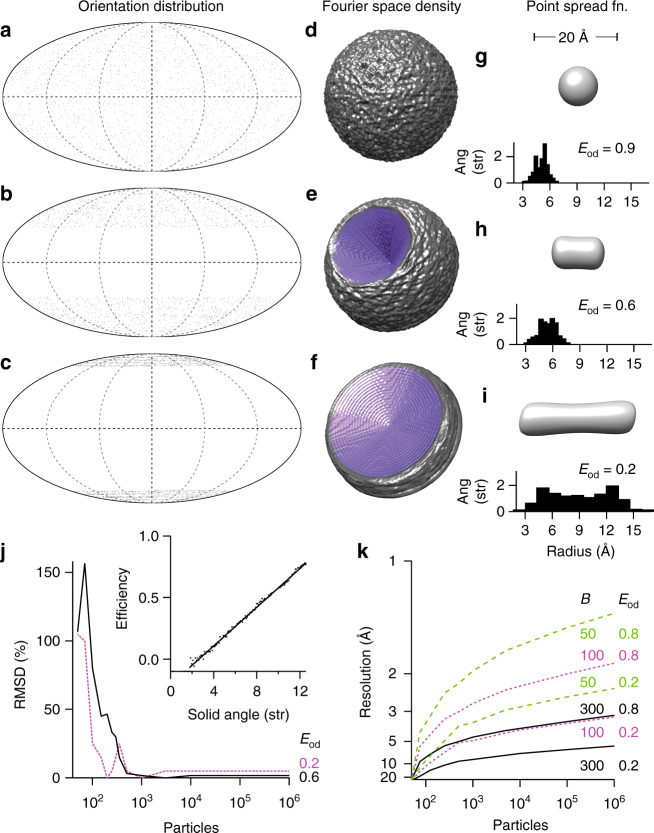
Particle orientation distributions can be described by their efficiency, *E*_od_. A uniformly distributed set of 10,000 particle orientations **a** provides uniform coverage of Fourier space **d**, which corresponds to a spherically symmetric PSF with a narrow distribution of radii **g** and high *E*_od_. A distribution with 1/3 of the views missing **b**, corresponds to a cone of missing density in Fourier space **e**, an elongated PSF with a larger variation in radii **h** and a reduced *E*_od_. A third distribution with 2/3 of the views missing (equiv. to a strongly preferred orientation) **c** corresponds to a large empty cone in Fourier space **f**, a strongly elongated PSF with a bimodal distribution of radii **i** and a low *E*_od_. The efficiency is linearly related to the fraction of Fourier space covered by the orientation distribution (**j**, *inset*). The root mean square error (RMSD) in efficiency is plotted vs. number particles used for the estimation **j**. The simulated relationship between the mean resolution and the number of particles used for a reconstruction is plotted **k** for different orientation distributions and *B*-factors

To determine how an orientation distribution affects 3D reconstructed density maps, we begin by assuming that each particle image is a two-dimensional projection of the electron scattering potential in that direction. By the central slice theorem, each projection can be represented by a plane of density in Fourier space. We further assume that the angle assigned to the particle in the 3D reconstruction process is correct to within a defined accuracy, and that the fall-off in information with resolution can be modelled as a Gaussian function characterised by a factor *B*, analogous to the Debye-Waller temperature factor used to describe the random thermal motion of atoms^9^. Given a distribution of particle angles (Fig. 1a–c), we can calculate the normalised information transmission in Fourier space (Fig. 1d–f), and the corresponding point spread function (PSF), (Fig. 1g–i). We then determine the radius along a particular direction where the normalised PSF falls to 1/*e* ^2^. The radii for all directions of several PSF’s calculated from the simulated distributions (Fig. 1a–c), are binned and plotted as histograms in Fig. 1g–i. Since the resolution in a particular direction is directly related to the radius of the PSF in that direction, we use this to define the efficiency as

1 $${E_{{\rm{od}}}} = 1 - \frac{{2\sigma }}{{\bar r}}$$

where $\bar r$ and *σ* are the overall mean and standard deviation of the resolution distribution, respectively. The *E*_od_’s for the distributions in Fig. 1 are indicated. We note that in principle the PSF can be used to deconvolve some of the distortions caused by the orientation distribution as has been done in other optical microscopy methods^10^. This is analogous to anisotropic sharpening used previously in electron microscopy^6, 7^, but deconvolution cannot compensate for a lack of information caused by large gaps in the Fourier space density map.

We used the above theoretical framework to create an algorithm to calculate the Fourier space density map, the PSF, the directions of highest and lowest resolution, and the efficiency from a given orientation distribution. It is implemented in a freely available, open source program called cryoEF. The program takes as input the Euler angles assigned to a particle set from any of the popular 3D reconstruction programs, including Frealign, EMAN and RELION^11–13^.

### Properties of the metric

We defined the efficiency, somewhat arbitrarily, to have four properties useful for data assessment. First, the efficiency is linearly related to the amount of solid angle absent in the Fourier space density map (Fig. 1j, *inset*). Second, the efficiency can be determined accurately with only a small number of particles (~1000, Fig. 1j and Supplementary Fig. 1), as long as a three-dimensional reconstruction can be computed with a reasonable angular accuracy. Third, a perfect orientation distribution would have an efficiency equal to 1, with a value of 0.5 being the approximate threshold between a distribution that will successfully reach high-resolution (>1/(4 Å)) and one that will not, using current microscope technology and a typical amount of data (~10^5^ particles). Fourth, the efficiency is specifically a property of the orientation distribution, and only weakly depends on other factors that can affect resolution like image quality (*B*-factor), specimen heterogeneity and data classification and processing algorithms. In this way it isolates one important factor in cryo-EM structure determination: the orientation distribution.

To understand how efficiency relates to other factors that bear on the ultimate resolution of a reconstruction, we calculate how the mean resolution scales with number of particles, for different values of *B* (image quality), and *E*_od_ (efficiency), by extending the theory of image contrast and resolution vs. particle number developed previously^9^. The loss of contrast at high resolution is modelled by a Gaussian fall-off in the recorded Fourier amplitudes and the resolution limit is determined by the intersection of the average amplitude with the noise level, which scales inversely with the root of the number of particles. The result is shown in Fig. 1k. First, it is clear that the image quality, here taken as the *B*-factor, is the most important determinant of the resolution achieved for the currently practical number of particle images in a data set (10^4^–10^6^ asymmetric views). The new generation of electron detectors improve image quality and, therefore, reduce the number of particles required to reach a particular resolution. Still, if there is insufficient Fourier space coverage, even a large data set will fail to reach high resolution, as indicated in Fig. 1k. Further, given a data set, one can use this theory to estimate the number of particles required to reach a particular resolution. Thus, one can rationally decide if it would be better to collect more data to reach a desired resolution or instead try to improve the efficiency through the use of specimen tilt or other specimen supports or preparation methods.

### Optimal tilt angles for data collection

We also include an algorithm for predicting tilt angles for data collection, which will improve the efficiency and the resolution of a data set. This is based on finding the minimum tilt angle that rotates the direction of highest resolution in the PSF to the direction of lowest resolution, and includes a model of contrast loss at tilt where the contrast is proportional to the cosine of the tilt angle. We demonstrate this improvement experimentally (Supplementary Fig. 2); while tilting the specimen can help in some instances, it cannot overcome the problems of reduced image quality at tilt, which is ultimately the more important factor in determining the resolution of a reconstruction (Fig. 1k). Tilting also cannot ameliorate the potentially destructive^5^ molecule–surface interactions indicated by an anisotropic orientation distribution. These can cause deformation or degradation of the specimen that cannot be compensated for in the imaging and reconstruction process.

### Efficiency of previously published data sets

**Fig. 2:**
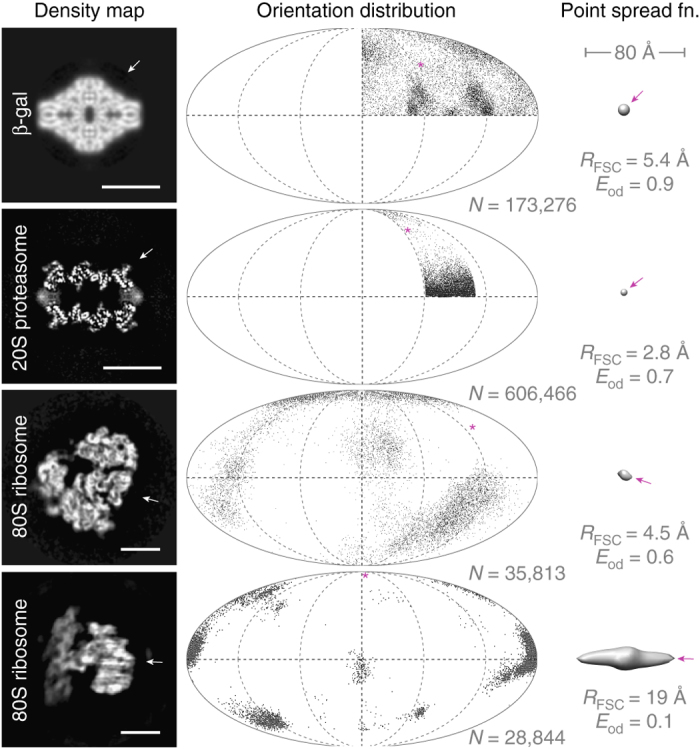
Assessment of the efficiency of particle orientation distributions in previously published data sets. Each *row*, from *left* to *right*, shows density map of specimen, orientation distribution of particles in the data set, corresponding point spread function and efficiency (*N* is the number of asymmetric units, *R*_FSC_ is the FSC resolution and *E*_od_ the efficiency). *Scale bars* are 100 Å. Incomplete orientation sampling causes distortion of the 3D density map, which can be described by the shape of the point spread function. The beta galactosidase and the 20S proteasome data sets have relatively uniform orientation distributions, which benefit from the symmetry of the particles, and correspondingly high efficiencies. Their PSFs are close to spherical, meaning that the resolution is isotropic. The two ribosome data sets, where the particles exhibit preferred orientations, have lower efficiencies and yield 3D reconstructions, which are smeared (severely, in the last case) in a particular direction, corresponding to the elongation of the point spread function in that direction. *Arrows* and *stars* indicate the direction of minimum resolution

To evaluate the algorithm, we tested it on four previously published data sets, which have orientation distributions with a wide range of uniformity (Fig. 2). The first distribution, that of the symmetric enzyme beta galactosidase (β-gal, D2 symmetry)^14^, was close to random and provided almost uniform coverage of Fourier space and a near spherical PSF (*E*_od_ = 0.9). Second, the distribution of a 20S proteasome specimen^15^, which has D7 symmetry, was assessed. Despite the fact that the vast majority of particles were oriented in one view (with the cylindrical symmetry axis parallel to the water surface), the efficiency was still high (*E*_od_ = 0.7) because of the symmetry of the complex. Third, an 80S ribosome data set on glow-discharged amorphous carbon^16^, which yielded a 4.5 Å reconstruction with a moderate number of particles was assessed. Although there were clearly several preferred orientations in the distribution, they were sufficiently separated to yield an oblate PSF with an efficiency of 0.6, which is less than optimal but still sufficient to reach high resolution. Fourth, a data set^17^ for the same 80S ribosome specimen (this time without the carbon–water surface), but having strong preferential orientations and insufficient sampling to reach high resolution (19 Å), was tested and had a correspondingly low efficiency (*E*_od_ = 0.1). Here, the particle oriented itself strongly at the air–water surface, causing a large gap in Fourier space, which limited the resolution to ~40 Å along the preferred direction.

## Discussion

The metric described here allows rapid and rigorous evaluation of specimen support materials and experimental conditions during sample preparation. This will enable one to reduce potentially detrimental surface interactions while optimising the orientation distribution for structure determination with minimal data. To demonstrate this, we collected a data set of approximately 2000 particle images (19 micrographs) of an 80S ribosome specimen, under identical conditions to those for the data in Supplementary Fig. 2, which allowed accurate determination of the efficiency of its orientation distribution. The entire process, from loading the grid in the microscope to reconstructing the density map and measuring the distribution and efficiency took 2 h. This shows that in one microscope day, one could potentially collect enough data to evaluate multiple different specimen support and preparation conditions, or quickly collect and process a test data set, which could then be used to guide data collection for the remainder of the session.

Surprisingly, the efficiency is as important as the amount of data in determining the resolution of a cryo-EM structure, and can be used to predict the amount of data needed to reach a particular resolution. The efficiency can be used to characterise the orientation distribution of any single-particle cryo-EM data set, and could be included with other metrics of data quality like Fourier shell correlation coefficients and local resolution^18, 19^ when reporting cryo-EM structures. Modern chromatography gels and resins comprise a myriad of specific surfaces that are carefully tuned to control molecule–surface interactions. An orientation distribution can be thought of as a map of the specimen’s surface interaction properties, and can thus be used to further our understanding of how biological molecules interact with specific surfaces. We thus envision that this statistical method will enable the further development of support surfaces like modified graphene^17^, specifically tuned to match the specimen under study.

## Methods

### Efficiency calculation algorithm

To assess the effects of the orientation distribution on the resulting three-dimensional reconstruction, we propose a definition of directional resolution. A unique point spread function corresponds to the geometry of any orientation distribution; we use the radius of this point spread function as a measure of directional resolution.

To obtain the point spread function for an arbitrary orientation distribution of projection images, the following steps are performed. We first calculate the transfer function, which corresponds to the coverage of Fourier space by the given distribution. If the molecule under consideration is known to be symmetric, we determine the orientation angles over all asymmetric units by applying the appropriate transformation matrices to the given distribution of views. By the central slice theorem, each two-dimensional projection image from a known direction of view (*ϕ*, *θ* in spherical coordinates) corresponds to a plane in three-dimensional Fourier space, perpendicular to this direction. Following Rosenthal and Henderson, we model the signal strength to decrease exponentially with *B*-factor dependence in Fourier space^9^:

$${C_i}({\bf{k}}) = \sqrt {{N_i}} {\rm{exp}}\left( { - \frac{{B{{\left| {\bf{k}} \right|}^2}}}{4}} \right)$$

Here *C*_*i*_ are the amplitude modulation coefficients, which describe how signal strength falls off at higher spatial frequencies for each different orientation (numbered *i*), *N*_*i*_ is the number of particles in this orientation, and **k** is a position vector, varying over the projection plane in three-dimensional Fourier space. The contributions of all projection views are then added up in quadrature, i.e., in power, rather than in amplitude, to give the three-dimensional Fourier space transfer function *τ*, imposed by the orientation distribution:

$$\tau ({\bf{k}}) = \sqrt {{\sum} {C_i}^2({\bf{k}})}$$

The transfer function is normalised to unit power. It represents the filter *τ*(**k**), with which the real object amplitude function *O*(**k**) is multiplied in Fourier space to yield the observed amplitudes of the image: *I*(**k**) = *O*(**k**) × *τ*(**k**). The corresponding real space PSF *s*(**r**) is obtained from the transfer function *τ*(**k**) by inverse Fourier transformation. By the convolution theorem, the three-dimensional image is then given by the convolution of the real object function with the PSF: *i*(**r**) = *o*(**r**) * *s*(**r**). Therefore, the radius of the point spread function at a given threshold value can be a measure of the resolution imposed by the geometry of the orientation distribution. Here, we choose the threshold value to be at the $\frac{1}{{{e^2}}}$ point relative to the central maximum, which corresponds to the point beyond which two Gaussian functions can no longer be separated.

The shape of the PSF can be used to quantitatively assess the underlying orientation distribution in terms of providing uniform Fourier space coverage, and hence isotropic resolution. By sampling the radius of the PSF in all directions, the spatial distribution of resolution is obtained. The relative spread of this distribution is directly related to the degree of anisotropy of the PSF. We define the efficiency of an orientation distribution, *E*_od_, as

$${E_{{\rm{od}}}} = 1 - \frac{{2\sigma }}{{\bar r}}$$

where $\bar r$ is the mean radius and *σ* is the standard deviation. For a perfectly uniform distribution, *E*_od_ = 1 since *σ* = 0, while for a single view *E*_od_ approaches zero or can even become slightly negative.

### Optimal tilt angle calculation algorithm

From the PSF we identify the weakest resolved directions in the map and determine tilt axes and angles, which can improve the resolution in these directions. By applying rotation matrices, we calculate how collecting data at tilt changes the apparent particle orientation angles, and, therefore, the Fourier space coverage and the real-space PSF shape and orientation. In practice, attainable tilt angles are limited by the quality loss in the resulting micrographs. We hypothesise that micrograph quality loss depends on the cosine of the tilt angle, i.e., the effective transverse thickness of the specimen. Due to this signal degradation, a tilt angle that improves the final resolution is not guaranteed to exist in all cases. We find the optimal tilt angle and axis (if these exist), which are expected to yield the most significant resolution improvement in the weakest direction.

### Software implementation and workflow

The algorithm described here is implemented in C++, using the publicly available library fftw3^20^ for Fourier transform computation on a three-dimensional grid. For a visual presentation of the results, the Fourier space coverage and the corresponding real-space PSF are written to MRC format (.mrc) binary files^21^. The point spread function can be conveniently displayed next to the corresponding density map to identify the weakest (most smeared) direction in the map.

For executing the program, a two-column list of the orientation angles of the particles in the format (*ϕ*, *θ*) is required. Software tools for de-novo model generation are readily available^22–24^ and can produce sufficient angular assignment accuracy to make efficiency estimates. Still, for most accurate results a fully converged 3D reconstruction is required. The other required input is an estimate of the object size *D* (in Å), which determines the smallest meaningful sampling step in Fourier space $\left( {\frac{1}{D}} \right)$. Other inputs are optional, and set to have appropriate default values. These include: FSC resolution, B-factor, box size, symmetry group, etc. The symmetry conventions we use are identical to those employed in XMIPP^25^ and RELION^13^. If the user inputs an FSC resolution, the algorithm scales the results accordingly. Typical computation time is of the order of 0.1–10 h, varying almost linearly with the size of the data set. This should not be a major limitation when using the method to analyse different experimental conditions, as small data sets (of ~1000 particles) are sufficient for assessment (Fig. 1j), and larger data sets can be fragmented into representative random subsets without introducing a significant error (Supplementary Fig. 2b).

### Data collection and analysis

The 80S ribosome data for Supplementary Fig. 1 was collected with a specimen prepared on an all gold specimen support, as previously described^26^. The use of gold supports reduces the radiation-induced specimen movement, the vast majority of which is perpendicular to the suspended foil, and whose transverse component severely compromises the quality of images collected at tilt. Briefly, 80S ribosome specimen was applied to a Ar:O_2_ (9:1) plasma treated all gold support without any additional layers, blotted, plunge frozen and imaged in a FEI Titan Krios at 300 keV under low-dose conditions. A small data set was collected (~ 3000 particles) and processed with RELION to obtain the orientation angles; the grid was stored during data processing. These were then used as input to the cryoEF program to asses the efficiency of the distribution and provide a predicted optimal tilt angle (29° for these data). A second small data set was then collected on another region of the same grid under the same conditions, at the prescribed tilt angle, and was analysed in the same way to generate the data for Supplementary Fig. 1.

### Data availability

The source code for the presented method, accompanied by a user manual, precompiled binaries, and a test data set are freely available under the terms of an open source software license at the authors’ website (http://www.mrc-lmb.cam.ac.uk/crusso/cryoEF/). Previously published data sets used for testing are available from the Electron Microscopy Data Bank (https://www.ebi.ac.uk/pdbe/emdb/) under accession codes 2548, 6287 and 2275. Supporting data are available from the corresponding author upon reasonable request.

## Electronic supplementary material

Supplementary Information
