# Supplementary material for: Measuring the effects of particle orientation to improve the efficiency of electron cryomicroscopy
Source: Nat Commun. 2017 Sep 20;8:629. doi: 10.1038/s41467-017-00782-3 (PMC5607000; doi:10.1038/s41467-017-00782-3)
Supplement: Supplementary file 1 — Supplementary Information [file 41467_2017_782_MOESM1_ESM.pdf]

Supplementary Note 1: On the origin of preferred orientations in single-particle cryo-EM

To good approximation, water and its saline forms in biology are isotropic over length scales ranging from ~ 10 Å to > 10 mm. So, excluding externally applied force fields, a solvated biomolecular complex will show no orientation preference when isolated in solution. It is only upon encountering a surface that it can be oriented in a non-random way with respect to the laboratory frame of reference. Cryo-electron microscopy requires a specimen that is as thin as possible to maximise image contrast. In practice, the ideal water layer is just thicker than the particle, typically anywhere from order 100 to 1000 Å.

Proteins can interact with surfaces by a number of different forces, which act at several length scales: from short-range interactions like hydrogen bonding and Van der Waals forces (~ 1 Å), to medium-range forces like the hydrophobic interaction (~ 10 Å), to long-range forces like Coulomb interactions ($\sim$ Debye length scale, 10 to 100 Å in 100 to 1 mM salt) [1].

Using this knowledge of the interaction length scales in the problem, we estimate to within an order of magnitude, how many times a particle may interact with a surface during the time between when the specimen is applied to the grid and excess water is removed and when it is rapidly frozen in a cryogen. If we take the mass of the protein particle in the range from 100 kDa to 1 MDa, then the corresponding diffusion constant, D, is of order 10^{-6} to 10^{-7} cm^{2}s^{-1} at 20°C [2], and scales according to $D \propto MW^{-1/3}$ [3]. The average distance Δx diffused in time t can be calculated as

$$\Delta x = \sqrt{2Dt} \tag{1}$$

The relevant time here is the residence time, i.e. the time elapsed between the removal of excess water to establish the thin film and the time at which it is frozen.

Supplementary Table 1: **Diffusion of molecules in a thin film of water**

MW (kDa)	D (cm^{2}s^{-1})	Residence time (s)	d (Å)	Num. short-range interactions	Num. hydrophobic interactions	Num. electrostatic interactions
10	1×10^{-6}	10	500	10^3	10^3	10^3
10^2	5×10^{-7}	10	500	10^3	10^3	10^3
10^3	2×10^{-7}	10	500	10^3	10^3	10^3
10^4	1×10^{-7}	10	2000	10^2	10^2	10^2
10	1×10^{-6}	1	500	10^3	10^3	10^3
10^2	5×10^{-7}	1	500	10^2	10^2	10^3
10^3	2×10^{-7}	1	500	10^2	10^2	10^2
10^4	1×10^{-7}	1	2000	10	10	10^2
10	1×10^{-6}	10^{-3}	500	10	10	10
10^2	5×10^{-7}	10^{-3}	500	10	10	10
10^3	2×10^{-7}	10^{-3}	500	10	10	10
10^4	1×10^{-7}	10^{-3}	2000	1	1	1

We perform calculations for the range of values relevant to cryo-EM specimens, and tabulate the results in Supplementary Table 1, where MW is the molecular weight, D is the corresponding diffusion constant, and d is the water layer thickness. To estimate the number of interactions we take diffusion path length

as the distance from the middle of the water layer to the point at which the corresponding interaction with the surface is relevant. Based on these order of magnitude estimates, we conclude that particles on cryo-EM specimen supports interact with the water surfaces anywhere from 10 to 1000 times during the grid preparation residence times of $1 - 10$ seconds. Even with an as yet to be created, ideal blotting and freezing instrument with a minimal residence time of ~ 1 ms, particle-surface interactions will still take place tens of times before the water is frozen. To eliminate these interactions one would need to further reduce the residence time by a factor of ~ 1000, such that the average number of interactions per particle is much less than 1. This means that the most promising approach to reduce preferred orientations is to control the surfaces present in the specimen support, since interaction with them is currently inevitable.

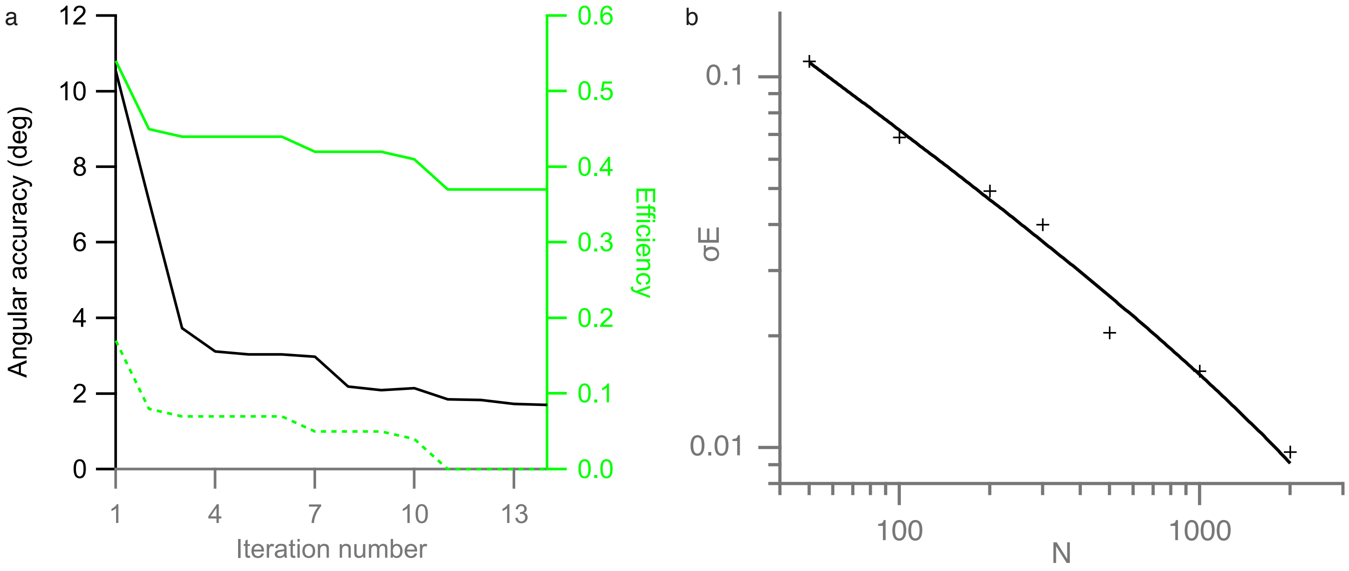

Supplementary Fig. 1: **The accuracy of the efficiency estimate depends on the angular assignment accuracy and is only weakly degraded when a large dataset is randomly downsampled.** (**a**) The efficiency estimate converges with the angular accuracy of the three-dimensional reconstruction. The efficiency calculated for the data from Supplementary Fig. 1a (green curve) and the accuracy of orientation angles assignment (black curve) are plotted against the iteration number of the three-dimensional structure refinement in RELION. An angular accuracy of 2-4 degrees is sufficient for efficiency estimation with an error of $< 10\%$. (**b**) The efficiency of the orientation distribution from Fig.2, row 3, is calculated with 10 random subsets of N particles and the standard deviation versus N is plotted (+ markers) on a log-log scale. The solid curve is a fit to the data using the model $\sigma_E = A + \frac{B}{\sqrt{N}}$. The dataset contains 35,813 views and the efficiency can be estimated with an error of $< 1\%$ from as few as 1,000 randomly selected particles.

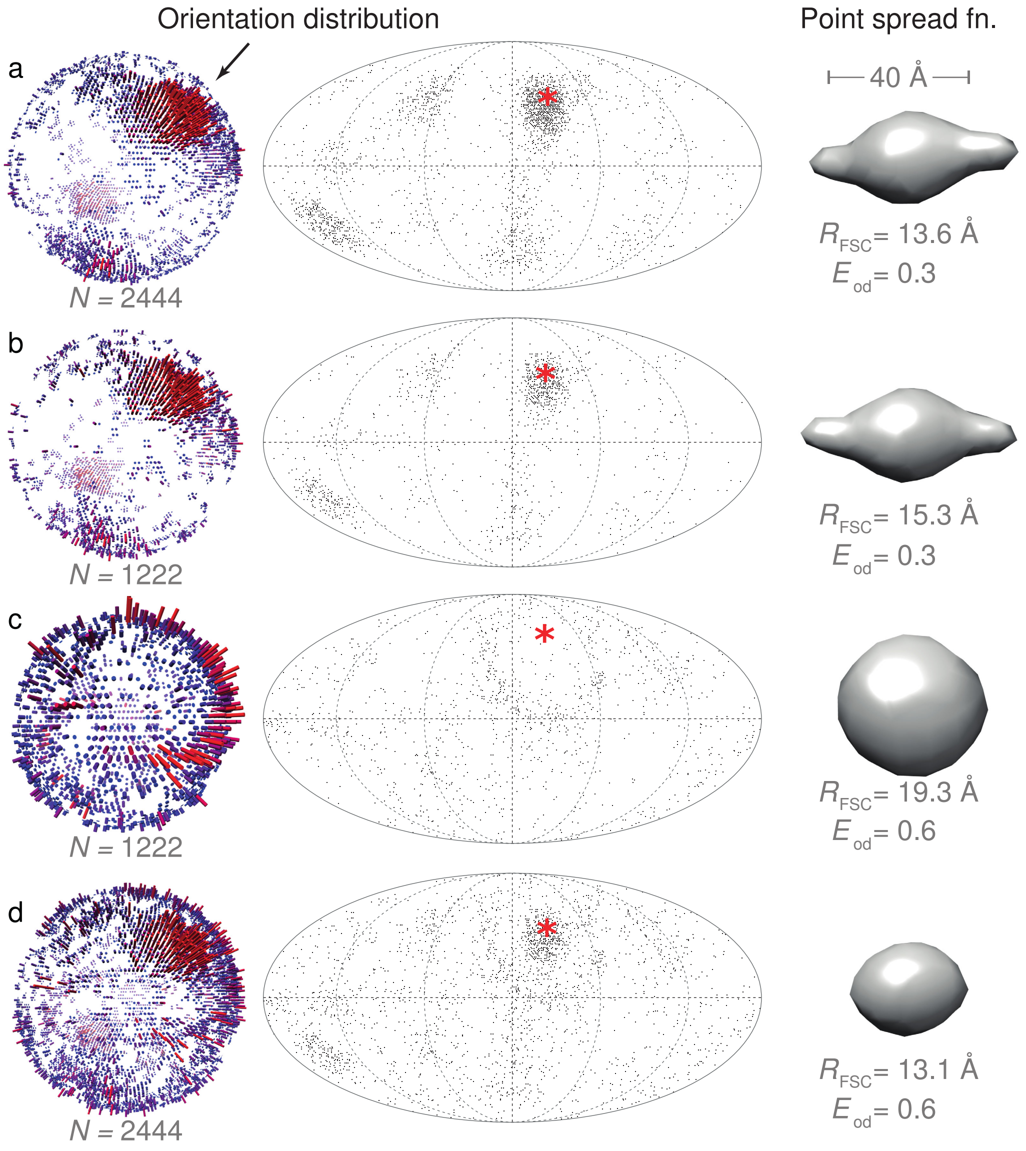

Supplementary Fig. 2: **Collecting data at an optimal tilt angle can improve the efficiency of an orientation distribution with a strongly preferred orientation.** Each row, from left to right, shows orientation distribution of particles on a sphere, orientation distribution on a Mollweide projection, corresponding point spread function and efficiency, FSC resolution at 0.143 as reported after 3D reconstruction. (**a**) Original dataset of 2444 ribosome particles imaged in ice with no additional support layer and without tilting. The particles show a strongly preferred orientation (indicated by an arrow), and hence the efficiency of the distribution is relatively low (0.3). The point spread function is elongated in the direction of the preferred orientation, corresponding to significantly worse resolution in that direction. (**b**) Randomly selected subset of 1222 particles from original dataset. Downsampling the orientation distribution does not affect its efficiency, but the reconstruction yields lower resolution due to halved number of particles. (**c**) Data collected at optimal tilt angle of 29°, as recommended by the algorithm. The strongly preferred orientation here results in a continuum of orientations arranged in a circle on the sphere, hence providing better Fourier space coverage and improved efficiency of 0.6. However, the resolution of the resulting reconstruction is worse compared to that in (b), due to the lower-quality micrographs obtained when tilting the specimen. The interplay of these two factors is taken into account when determining the optimal tilt angle. (**d**) Data from (b) and (c) is combined to give a dataset of 2444 particles - the same size as in (a), but with half of the particles imaged at tilt. This combination improves both the efficiency and the resolution, relative to (a). The improvement in the resolution is due to the improved efficiency, with the number of particles being the same as in (a), and the quality of the micrographs as in (b) and (c). The FSC resolution is improved by virtue of partially eliminating the weakly resolved direction, as shown in the shape of the point spread function.

References

[1] Israelachvili, J. N. *Intermolecular and Surface Forces* (Academic Press, 2011), 3 edn.

[2] Brune, D. & Kim, S. Predicting protein diffusion coefficients. *Proc. Natl. Acad. Sci. USA* **90**, 3835–3839 (1993).

[3] Landau, L. D. *Fluid Mechanics (Course of Theoretical Physics)*, vol. 6 (Butterworth-Heinemann, 1987), 2 edn.
